# Towards evidence-based practice 2.0: leveraging artificial intelligence in healthcare

**DOI:** 10.3389/frhs.2024.1368030

**Published:** 2024-06-11

**Authors:** Per Nilsen, David Sundemo, Fredrik Heintz, Margit Neher, Jens Nygren, Petra Svedberg, Lena Petersson

**Affiliations:** ^1^ School of Health and Welfare, Halmstad University, Halmstad, Sweden; ^2^ Department of Health, Medicine and Caring Sciences, Linköping University, Linköping, Sweden; ^3^ School of Public Health and Community Medicine, Institute of Medicine, Sahlgrenska Academy, University of Gothenburg, Gothenburg, Sweden; ^4^ Lerum Närhälsan Primary Healthcare Center, Lerum, Sweden; ^5^ Department of Computer and Information Science, Linköping University, Linköping, Sweden

**Keywords:** artificial intelligence, evidence-based practice, clinical decision-making, evidence, clinical experience, patient preferences

## Abstract

**Background:**

Evidence-based practice (EBP) involves making clinical decisions based on three sources of information: evidence, clinical experience and patient preferences. Despite popularization of EBP, research has shown that there are many barriers to achieving the goals of the EBP model. The use of artificial intelligence (AI) in healthcare has been proposed as a means to improve clinical decision-making. The aim of this paper was to pinpoint key challenges pertaining to the three pillars of EBP and to investigate the potential of AI in surmounting these challenges and contributing to a more evidence-based healthcare practice. We conducted a selective review of the literature on EBP and the integration of AI in healthcare to achieve this.

**Challenges with the three components of EBP:**

Clinical decision-making in line with the EBP model presents several challenges. The availability and existence of robust evidence sometimes pose limitations due to slow generation and dissemination processes, as well as the scarcity of high-quality evidence. Direct application of evidence is not always viable because studies often involve patient groups distinct from those encountered in routine healthcare. Clinicians need to rely on their clinical experience to interpret the relevance of evidence and contextualize it within the unique needs of their patients. Moreover, clinical decision-making might be influenced by cognitive and implicit biases. Achieving patient involvement and shared decision-making between clinicians and patients remains challenging in routine healthcare practice due to factors such as low levels of health literacy among patients and their reluctance to actively participate, barriers rooted in clinicians' attitudes, scepticism towards patient knowledge and ineffective communication strategies, busy healthcare environments and limited resources.

**AI assistance for the three components of EBP:**

AI presents a promising solution to address several challenges inherent in the research process, from conducting studies, generating evidence, synthesizing findings, and disseminating crucial information to clinicians to implementing these findings into routine practice. AI systems have a distinct advantage over human clinicians in processing specific types of data and information. The use of AI has shown great promise in areas such as image analysis. AI presents promising avenues to enhance patient engagement by saving time for clinicians and has the potential to increase patient autonomy although there is a lack of research on this issue.

**Conclusion:**

This review underscores AI's potential to augment evidence-based healthcare practices, potentially marking the emergence of EBP 2.0. However, there are also uncertainties regarding how AI will contribute to a more evidence-based healthcare. Hence, empirical research is essential to validate and substantiate various aspects of AI use in healthcare.

## 1 Introduction

More than three decades ago, evidence-based medicine (EBM) emerged as a ground-breaking concept introduced by the Evidence-Based Medicine Working Group, marking a pivotal shift in medical practice (1). Originating at McMaster University in Hamilton, Canada, the term was coined to describe a new methodology in learning the practice of medicine (2). EBM was developed in response to the recognition of limitations and shortcomings in traditional medical practices, which often relied on anecdotal evidence, expert opinion and historical practices rather than rigorous scientific evidence. EBM emerged as a response to the need for a more systematic and scientific approach to medical decision-making. It aimed to improve the quality of patient care by ensuring that medical interventions and treatments were based on sound empirical evidence and demonstrated effectiveness through rigorous evaluation methods such as randomized controlled trials (RCTs) (3).

Subsequently, EBM swiftly disseminated and its influence expanded to become evidence-based practice (EBP), extending beyond traditional medical domains to disciplines such as nursing, mental health, physiotherapy, occupational therapy, as well as broader fields such as public health, social work, education, and management. At the same time, a vertical spread occurred from an early focus on various forms of interventions to also include the policy process concerning the use of evidence for identifying and prioritizing problem areas and for decision-making (4). This widespread adoption was facilitated by technological advancements, including electronic databases and the Internet (5).

EBP is often described in terms of decisions being made based on three sources of information: evidence, clinical experience and patient preferences (6). However, despite the popularization of EBP, research in implementation science has shown that there are many barriers to achieving the goals of the EBP model, including practitioners having insufficient time to find relevant studies or guidelines and a lack of skills and confidence in assessing the quality of research. There are also organizational barriers such as lack of leadership support and a culture that does not facilitate the use of research in practice (7).

The use of artificial intelligence (AI) in healthcare has been proposed as a means to improve clinical decision-making (8–12). The upsurge in interest in AI, which involves machines or computer systems simulating human intelligence processes, has been propelled by the exponential increase in computing capabilities and the abundance of available healthcare data (8–12). Under the definition approved by the EU Parliament, an AI system means “a machine-based system designed to operate with varying levels of autonomy, that may exhibit adaptiveness after deployment and that, for explicit or implicit objectives, infers, from the input it receives, how to generate outputs such as predictions, content, recommendations, or decisions that can influence physical or virtual environments” (13). The promise of AI has fostered expectations of a paradigm shift towards an “AI-assisted” (also referred to as “data-driven” or “information-driven”) healthcare, with expectations of improving care quality while curbing costs (14, 15). This review pinpoints key challenges pertaining to the three pillars of EBP: evidence, clinical experience and patient preferences. The potential of AI to surmount these challenges and contribute to a more evidence-based healthcare practice is discussed.

A selective review of the literature on EBP and the integration of AI in healthcare was undertaken, focusing on the domains of evidence, clinical experience and patient preferences. Thus, this article draws on secondary sources, encompassing empirical studies, overviews, reviews, assessments of research and opinion papers. Although the primary responsibility for sourcing and evaluating pertinent literature rested with the first author (PN), all authors collaborated in identifying relevant materials for this review.

## 2 Challenges with the three components of EBP

This section addresses the challenges identified in research associated with the three key components of EBP: evidence, clinical experience and patient preferences.

### 2.1 Challenges concerning evidence

The EBP model is based on the existence and availability of evidence to inform clinicians' decision-making to achieve evidence-based healthcare. Nevertheless, there are numerous hurdles in the transition from the generation of evidence to its practical use. The journey from research conception and publication to dissemination and implementation in routine healthcare is often dogged by significant time gaps. The prolonged duration from inception to publication may render the findings outdated upon release. Furthermore, the scarcity of high-quality evidence stemming from RCTs, which is the pinnacle in the evidence hierarchy, compels clinicians to rely on lower-tier sources of evidence. In several instances, the absence of any evidence compounds these challenges, leaving practitioners with no foundational support (16, 17).

Systematic reviews constitute a cornerstone of the EBP model. These reviews locate, assess and synthesize evidence on a health topic, making the information accessible and digestible to clinicians and decision-makers. They also serve as the foundation for developing clinical guidelines (18). However, the process of generating evidence via systematic reviews is typically slow and resource-intensive, involving substantial time for preparation and writing. Factors such as the authors’ expertise, methodologies used and the number of studies incorporated contribute to this duration (19). Studies have indicated that the average duration to complete a systematic review exceeds 15 months (20). Consequently, there is a risk that once published, a systematic review may already be outdated (18). Moreover, due to the labour-intensive nature of this process, many systematic reviews are not updated at sufficiently regular intervals (21).

### 2.2 Challenges concerning clinical experience

Clinicians gain their expertise through many years of training and practice, from undergraduate education to higher degrees and hands-on experience in real-world healthcare settings. This extensive training is essential to ensure clinicians possess the proficiency required for accurate diagnosis, treatment and prognosis for their patients (22). Clinical experience equips clinicians with the ability to draw informed conclusions about patients' health conditions, enabling them to make critical assessments, distinctions and decisions based on their accumulated knowledge. Although the EBP model underscores the significance of evidence in clinical decision-making, its direct application is not always feasible. Clinicians must leverage their clinical experience to interpret the relevance of research findings and contextualize them within the unique needs and conditions of their patients (17).

Clinical experience plays a crucial role in discerning the applicability of research-generated evidence to individual patients, primarily because evidence is often derived from studies involving patient groups that differ from those encountered in routine healthcare. These studies typically involve homogeneous populations, unlike the diverse range of individuals seen in everyday practice. As a result, patients in routine healthcare often exhibit variations from the averaged descriptions found in research (23). Research conclusions hold validity at a population level, but they might not necessarily apply uniformly at the individual patient level (4). Consequently, even if the evidence supports a specific treatment for a particular patient population, clinical experience becomes essential in gauging the extent to which this evidence can be extended and tailored to suit the needs of specific individuals.

Clinical experience can be susceptible to various cognitive biases, i.e., distortions in judgement and decision-making arising from the human brain's inclination to simplify and expedite information processing based on personal experiences and preferences (24). These biases have the potential to significantly affect clinical decision-making, leading to adverse health outcomes (25). Studies have identified that time constraints, a hectic work environment, frequent task switching and the pressure to make swift decisions based on limited information contribute to high cognitive loads and work-related stress, further exacerbating the impact of these biases (26, 27). A review by Gopal et al. (25) identified 12 different types of cognitive biases in healthcare. For example, selection bias might occur when selective observations of favourable outcomes attributed to a certain treatment yield undue confidence in its effectiveness (28). Availability bias might occur as a result of having experience with cases or studies that come more easily to mind, which may yield assumptions of the same scenario being repeated (29). Further, satisfaction bias occurs when a clinician concludes a diagnosis when identifying a single disease as the root cause although more causes may be possible (30).

Clinical experience can also be influenced by implicit biases, i.e., the subconscious tendencies for stereotype-affirming thoughts to traverse our minds, potentially leading to discrimination (27). Implicit biases are associated with underlying attitudes, whether favourable or unfavourable, whereas cognitive biases are linked to thought processes (31). These implicit biases might be directed towards various characteristics such as race, gender, age or health conditions, possibly resulting in discriminatory practices. For instance, research indicates that healthcare professionals, including physicians, nurses, nutritionists and dietitians, often harbour biases against individuals with obesity. This prejudice leads to implicit assumptions regarding weight-related issues, associating them with a perceived lack of willpower or personal character (25, 32).

### 2.3 Challenges concerning patient preferences

The EBP model underscores the significance of patient preferences alongside evidence and clinical expertise. Patient engagement has long been seen as the “last mile” problem of healthcare, the assumption being that the more patients participate in their own care, the better the health outcomes (8). However, patient involvement and shared decision-making where clinicians and patients make decisions together have been found to be difficult to achieve in routine healthcare practice (33, 34). Multiple obstacles hinder this process, ranging from low levels of health literacy among patients and their reluctance to actively participate, to barriers rooted in clinicians' attitudes, scepticism towards patient knowledge and ineffective communication strategies. In addition, high workloads, bustling healthcare environments and limited resources pose further challenges (35, 36). Consequently, living up to the EBP model's aspiration of integrating patients' preferences into clinical decision-making often proves challenging.

## 3 Enhancing EBP through AI support

This section explores how the use of AI in healthcare can address challenges within the EBP model and enhance the three key components of evidence, clinical experience and patient preferences through AI support.

### 3.1 Enhancing evidence through AI support

AI presents a promising solution to address several challenges inherent in the research process, from conducting studies, generating evidence, synthesizing findings, and disseminating crucial information to clinicians to implementing these findings into routine practice. Notably, it has been suggested that AI may free up researchers' time to delve into new areas of pioneering research, commonly referred to as “blue skies” research (37). AI is proficient in focused tasks, such as sifting through abstracts to identify pertinent information and excels in analysing substantial volumes of unstructured data (38). Further, AI may assist in the laborious process of performing systematic reviews, increasing the relevance of the suggested articles, thereby saving valuable time (39). Consequently, AI can enhance researchers' efficiency, expediting the process of generating evidence.

AI presents an opportunity to enhance the efficiency and effectiveness of clinical RCTs, often considered the gold standard for generation of evidence. Its potential covers all phases of these trials, from discovery, pre-trial planning and design to patient recruitment, trial execution and analysis of results (40, 41). In pharmaceutical research, the discovery and design of potential drugs demand significant time and resources, often relying on labor-intensive processes (42). Leveraging AI-driven tools like Alphafold, researchers can predict the three-dimensional structure of amino acids, expediting the identification of promising compound candidates during the preclinical phase (41, 42). Moreover, AI can forecast potential toxicity and side effects in the initial stages, thus enhancing the likelihood of trial success. It is estimated that approximately 30% of potential drugs are discarded due to toxicity concerns, making AI-enabled toxicity prediction a valuable resource-saving tool (43). Additionally, AI can streamline the identification of suitable trial participants, as demonstrated by Hassanzadeh et al., who utilized an algorithm to match individuals with the eligibility criteria of relevant trials using text-based data extracted from patient records (44). During the active trial phase, AI offers improved patient monitoring, enhancing adherence to the medication or treatment regimen under study (45). AI systems can contribute to the analysis of the results by providing more comprehensive assessments, such as identifying key risk factors, managing missing data and automating data extraction to minimize human error. Moreover, AI's efficiency in supporting clinical trials expands the feasibility of conducting trials in areas where profitability might otherwise hinder progress, such as developing medications for rare diseases or targeted therapies (40).

AI can also play an important role in enhancing the synthesis of evidence within systematic reviews, reducing time and costs while enhancing efficiency (46). Notably, various AI systems can facilitate screening of titles and abstracts in systematic reviews (39). A systematic review of AI-assisted systematic reviews concluded that several AI systems “have taken hold with varying success in evidence synthesis” (47). This trend indicates a growing probability of AI-assisted systematic reviews gaining prevalence, thereby accelerating the synthesis of evidence. Furthermore, AI provides assistance in conducting literature reviews, particularly those that are less structured compared with quantitative systematic reviews (48).

However, AI is not without limitations when used for research purposes. Large language models (LLMs) may tempt researchers to utilize them for tasks beyond their validation, as their results often appear sound superficially (49). This poses challenges in qualitative evidence synthesis, which relies on careful consideration of evidence quality and quantity (50). For instance, summarizing evidence in a systematic review may overlook actual probabilities if LLMs generate text without causal understanding. If the training data for the LLM model is pertinent to the topic, the generated text may correlate with the underlying evidence, but it does not establish a causal relationship. Conversely, if the training data is irrelevant, the resulting text may be eloquent and persuasive but likely lacks accuracy and truthfulness (51). As LLM availability grows, there is a risk of diluting valid evidence with AI-generated content. Vigilance within the scientific community is crucial to prevent this.

Although AI offers support to researchers throughout various phases of the research process, integrating this evidence into clinical practice poses challenges for clinicians. They might encounter difficulties in critically reviewing and interpreting research findings or assessing the quality of the evidence, potentially due to limited training in developing academic evidence assessment skills (40, 45). Keeping up with the evolving research landscape requires significant time and expertise. Notably, Hoffmann et al.'s study (52) observed a drastic increase, more than 20-fold, in indexed systematic reviews in the past two decades, averaging 80 publications per day in 2019. Despite suggestions indicating AI's potential to afford clinicians more time to engage with evidence (33), empirical studies supporting this relationship are currently lacking.

### 3.2 Enhancing clinical experience through AI support

AI systems have a distinct advantage over human clinicians in processing specific types of data and information. Equipped with access to vast amounts of data and the capability to process them in real time, AI systems demonstrate an unparalleled capacity for rapid learning and adaptation, continuously enhancing their performance at an exponential rate (22). The use of AI in clinical decision-making offers clear benefits, notably in minimizing variations among clinicians, thereby ensuring more uniform and precise diagnoses, treatments and prognoses (53).

AI has shown significant promise in revolutionizing image analysis within healthcare. For example, Cohen et al. (54) showcased a recent study where an AI algorithm specialized in wrist fracture detection outperformed radiographic analysis conducted by non-specialized radiologists. The study highlighted that a combination of AI and physician analysis achieved the highest sensitivity in identifying radiographic fractures. This trend was echoed in a mammography study (55), where the combined approach of AI and physician assessment surpassed the performance of two individual physicians or AI analysis alone. In addition, a separate study (56) revealed that AI-supported mammography screenings achieved cancer detection rates comparable with readings by two physicians and the workload associated with screen reading was reduced considerably. Thus, clinicians using AI-driven clinical decision support systems are not confined by their own clinical experience but can harness data from thousands of relevant cases to enhance their decision-making process.

AI holds the promise of augmenting clinicians' capabilities and enriching their clinical experience through innovative simulation training methods. The emergence of LLMs presents an added avenue for creating interactive medical simulation cases. These simulations provide patients' responses to every conceivable action or reaction by medical students, offering promising educational possibilities (57). Recent studies using the LLM ChatGPT have demonstrated its efficiency and accuracy in simulating patients for educational purposes (58). Research is important to assess the equivalency of AI-simulated medical training to genuine patient consultations and to ascertain the extent to which the knowledge acquired from simulated cases translates into practical real-world healthcare scenarios (59).

AI may alleviate some of the challenges with clinicians' cognitive and implicit biases but AI systems still carry a risk for various flaws (60). For example, the input or output of an AI application may necessitate human judgement, such as a clinician determining what data should be used in the application (61). A novel challenge for clinicians is to know when and how to use the output from a particular algorithm in a clinical situation. The importance of increased knowledge among clinicians has been emphasized to ensure appropriate use of AI (62). Bias can be generated across AI system development, from the preparation and collection of the data to the development and training of the algorithm, and the evaluation and deployment of the system in clinical settings (15, 63). These challenges underscore the importance of not taking the objectivity of AI for granted but rather devoting research to investigate the consequences of AI flaws prior to routine use.

The journey towards expertise for clinicians is multifaceted, encompassing experiential learning and self-reflection, encountering errors, observing peers and receiving feedback from both senior staff and patients. Typically, clinicians start with basic tasks that require fundamental skills, gradually progressing to more complex responsibilities as their expertise advances. This gradual ascent builds a crucial foundational framework for expertise (64). However, the integration of AI in performing tasks traditionally conducted by clinicians may potentially omit the foundational learning stage acquired through years of practice. This transfer of responsibilities to AI could lead to deskilling, characterized by reduced clinician discretion, autonomy, decision-making capabilities and domain knowledge within their roles (65).

### 3.3 Enhancing patient preferences through AI support

AI presents promising avenues for enhancing patient engagement, aligning with the aspirations of the EBP model. Despite the lack of dedicated research on AI systems explicitly designed for this purpose, its capacity to save time for clinicians has been acknowledged, potentially fostering more meaningful interactions between clinicians and patients (8, 33, 66, 67). Previously, simpler algorithms like the Wells score for diagnosing deep vein thrombosis (68) and the FRAX score for assessing osteoporosis risk (69) have supported clinicians in medical decision-making. These tools help avoid unnecessary diagnostic procedures by identifying patients who are more likely to benefit. More advanced AI algorithms, like voice recognition and generative AI, have even greater potential to save clinician time by automating the transcription of complete clinician-patient interactions (70). Other examples include automating administrative tasks such as appointment scheduling, reminders and managing no-shows can alleviate clinicians' burden of data entry, freeing up time (71). Research on AI in radiology suggests that radiologists, with a reduced administrative load, can devote more time to patients, enabling them to prioritize personalized care (72).

AI holds promise in enhancing patient autonomy, presenting the prospect of a more balanced clinician-patient relationship with a more equitable decision-making process (73). For example, an AI-driven self-monitoring device has been developed to anticipate exacerbation risks (severe worsening of lung symptoms) in patients with chronic obstructive pulmonary disorder (COPD) (74). This device utilizes both hardware for biometric data capture and AI-driven software to predict exacerbation risks within the patient's home, eliminating the need for consultation with a clinician. Its goal is to offer automated recommendations to patients, empowering them to make informed decisions without the necessity of consulting a clinician.

While there is hope that AI will enhance patient autonomy, there are also uncertainties about how this will happen. Efficient algorithms require vast amounts of data, often sensitive health information in healthcare settings. Developing AI systems using non-anonymized data poses privacy risks, as individual patient data could be traced back (75). Additionally, AI-driven profiling may unveil health-related details about individuals without their consent. For instance, companies can predict individuals' health using publicly available social media data, potentially selling this information for profit. The conventional clinician-patient dynamic is often marked by the patient's vulnerability to the clinician, which raises questions concerning how AI might reshape this power balance. Empirical questions remain on the impact and evolution of the clinician-patient relationship with AI deployment, necessitating further investigation. In addition, accommodating diverse preferences in AI systems poses a challenge. McDougall (76) has raised a concern that the integration of AI might unintentionally promote a new type of paternalism, encouraging a paradigm where “the computer knows best”. Such a transition could contradict the recognized significance of incorporating the patient's viewpoint within the EBP model.

## 4 Discussion

Striving to achieve evidence-based healthcare practice aligned with the EBP model has encountered substantial challenges, resulting in the rapid growth of implementation science. This discipline aims to identify barriers and formulate strategies that enable the integration of research-based practices into routine healthcare (77). When evidence is beset with quality limitations, restricted applicability or even absence, clinicians' experience is necessary to contextualize and apply it in specific cases. In addition, clinical decision-making may suffer from cognitive and implicit biases, limiting its accuracy. Incorporating patients’ preferences poses another challenge within the EBP model. Recognizing these limitations, several scholars (8–12) advocate for leveraging AI to enhance clinical decision-making.

Our analysis underscores the potential of AI to enhance various aspects of the three pillars of EBP. Nonetheless, the use of AI comes with inherent limitations. There is a risk of perpetuating biases and the potential deskilling of clinicians as the automation of tasks progresses. Furthermore, within various AI applications in healthcare, we face a realm of uncertainty concerning its uncharted effects, leading to speculation due to the nascent stage of AI development and implementation in practice. Undoubtedly, the ongoing discourse on AI in healthcare will persist, necessitating empirical research to comprehend and shape its implementation and influence within healthcare.

AI has been lauded as a substantial time-saving tool, potentially affording clinicians more time to enhance their expertise in evidence assessment or deepen patient engagements (33). Nevertheless, skeptics have expressed concerns regarding the potential increase in the number of patients navigating the healthcare system due to economic dynamics (78). The actual outcome, whether AI will predominantly optimize throughput or pursue alternate objectives, will be contingent upon how healthcare systems and decision-makers prioritize efficiency relative to other crucial values. It is essential to study both the intended and unintended consequences of AI deployment in healthcare to gain a holistic understanding of its impact. A European survey by the European Patients' Forum highlighted the promise of AI in delivering more personalized care to patients (79), but further research is crucial to explore how AI implementation will influence clinician-patient relationships and its broader impact on enhancing patient involvement.

AI has faced criticism for its “black box” nature, making it challenging to decipher or explain the reasoning behind specific predictions or decisions due to the intricate structures and numerous variables within AI systems (80). Conversely, the EBP model was designed to elucidate clinical decision-making processes, ensuring transparency in understanding why certain decisions were made (2). Nevertheless, proponents argue that if AI consistently outperforms clinicians, the necessity for explainability diminishes (33). Thus, the credibility and trustworthiness of AI systems could be assessed based on the reliability of their output rather than the transparency of their processes. Moreover, it could be contended that traditional clinical decision-making also harbours “black box” components, where cognitive and implicit biases influence clinicians' decisions (81).

Another debate surrounding AI is the potential deskilling of clinicians due to the automation of tasks by AI systems. There are concerns regarding potential negative consequences, such as compromised decision-making, a decline in clinical skills and a possible compromise in patient safety (64). In the short term, the challenge of deskilling may not present a significant issue because the current clinical workforce has substantial clinical experience, making them valuable resources in handling complex cases that AI systems might find challenging. However, in the long term, newly educated clinicians may lack experience and proficiency in tasks that have been automated, potentially affecting the quality of care. Deskilling is not a new phenomenon; across various sectors, technological advancements have reduced the skill requirements for specific jobs over generations (82). The evolution and implications of deskilling in healthcare remain uncertain and warrant thorough investigation.

The ongoing debate on AI-induced deskilling resonates with the historical discourse that surrounded the emergence of the EBP model. Initially, the EBP model faced critique and was labelled as “cookbook medicine” and a “straitjacket” for clinicians. Concerns about potential de-professionalization arose because it was perceived that clinicians might lose their autonomy and critical judgement by adhering to pre-established guidelines and protocols (4, 83). Yet, while past criticisms have waned over time, the current discourse on the potential deskilling of clinicians due to AI automation seems more serious. This elevated gravity likely arises from heightened concerns about the possible displacement of the workforce by AI-enabled automation (64).

This selective literature review has some limitations that require consideration. The primary focus was to explore AI's potential impact on the three components of EBP: evidence, clinical experience and patient preferences. Consequently, the review did not encompass the extensive array of existing or potential applications of AI in healthcare, such as its use as a managerial tool for administrative tasks, resource allocation in healthcare facilities or facilitating continuous quality improvement initiatives through data analysis and feedback mechanisms. This review does not provide a comprehensive overview but rather focuses on AI in relation to the three pillars of EBP.

## 5 Conclusion

This review of the literature on EBP and AI in healthcare suggests considerable potential for AI to advance evidence-based healthcare practices, potentially heralding the advent of what might be termed EBP 2.0. Nonetheless, empirical research is crucial to substantiate various aspects of the use of AI in healthcare. There is speculation about AI potentially replacing clinicians' roles in healthcare, but we believe that human clinicians will continue to provide critical value for patients through their uniquely human attributes. Consequently, a paradox arises whereby the integration of AI might indirectly emphasize and increase the value of human skills within healthcare.
